# Predicting bleeding risk in a Chinese immune thrombocytopenia (ITP) population: development and assessment of a new predictive nomogram

**DOI:** 10.1038/s41598-020-72275-1

**Published:** 2020-09-18

**Authors:** Mingjing Wang, Weiyi Liu, Yonggang Xu, Hongzhi Wang, Xiaoqing Guo, Xiaoqing Ding, Richeng Quan, Haiyan Chen, Shirong Zhu, Teng Fan, Yujin Li, Xuebin Zhang, Yan Sun, Xiaomei Hu

**Affiliations:** 1 Xiyuan Hospital, China Academy of Chinese Medical Sciences, No.1 Xiyuan Caochang, Haidian District, Beijing, 100091 China; 2 Graduate School, China Academy of Chinese Medical Sciences, No. 16 Nanxiao Street, Dongzhimen, Dongcheng District, Beijing, 100700 China; 3 Graduate School, Beijing University of Chinese Medicine, No. 11 Bei San Huan Dong Lu, Chaoyang District, Beijing, 100029 China; 4 Dongfang Hospital, Beijing University of Chinese Medicine, No. 6 FangXingYuan 1st Block, Fengtai District, Beijing, 100078 China

**Keywords:** Cytokines, Inflammation, Diseases

## Abstract

The aim of this study was to develop a model that could be used to forecast the bleeding risk of ITP based on proinflammatory and anti-inflammatory factors. One hundred ITP patients were recruited to build a new predictive nomogram, another eighty-eight ITP patients were enrolled as validation cohort, and data were collected from January 2016 to January 2019. Four demographic characteristics and fifteen clinical characteristics were taken into account. Eleven cytokines (IFN-γ, IL-1, IL-4, IL-6, IL-8, IL-10, IL-17A, IL-22, IL-23, TNF-α and TGF-β) were used to study and the levels of them were detected by using a cytometric bead array (CBA) human inflammation kit. The least absolute shrinkage and selection operator regression model was used to optimize feature selection. Multivariate logistic regression analysis was applied to build a new predictive nomogram based on the results of the least absolute shrinkage and selection operator regress ion model. The application of C-index, ROC curve, calibration plot, and decision curve analyses were used to assess the discrimination, calibration, and clinical practicability of the predictive model. Bootstrapping validation was used for testing and verifying the predictive model. After feature selection, cytokines IL-1, IL-6, IL-8, IL-23 and TGF-β were excluded, cytokines IFN-γ, IL-4, IL-10, IL-17A, IL-22, TGF-β, the count of PLT and the length of time of ITP were used as predictive factors in the predictive nomogram. The model showed good discrimination with a C-index of 0.82 (95% confidence interval 0.73376–0.90 624) in training cohortn and 0.89 (95% CI 0.868, 0.902) in validation cohort, an AUC of 0.795 in training cohort, 0.94 in validation cohort and good calibration. A high C-index value of 0.66 was reached in the interval validation assessment. Decision curve analysis showed that the bleeding risk nomogram was clinically useful when intervention was decided at the possibility threshold of 16–84%. The bleeding risk model based on IFN-γ, IL-4, IL-10, IL-17A, IL-22, TGF-β, the count of PLT and the length of time of ITP could be conveniently used to predict the bleeding risk of ITP.

## Introduction

Immune thrombocytopenia (ITP) is a hematological disorder characterized by a decrease in platelet numbers with or without potential bleeding at multiple sites^1^. The destruction of the platelets and an increasing risk of bleeding have a close connection with immune disorder in T cells in ITP patients^2^. However, T helper (Th) cells compose a broad group of immune cells that includes T helper 1 (Th1) cells, T helper 2 (Th2) cells, T helper 17 (Th17) cells, and T regulatory (Treg) cells. Th1/Th2 and Th17/Treg cell imbalances have been confirmed in ITP patients^3,4^. Additionally, the imbalances between these T cells result in an inflammatory state, which contributes to vascular endothelial injury and platelet destruction in ITP patients. In addition, the differentiation of Th1 cells is induced by the cytokine IFN-γ, the differentiation of Th2 cells is induced by the cytokine IL-4, the differentiation of Th17 cells is induced by the cytokine IL-17, and the differentiation of Treg cells is induced by the cytokine TGF-β. The activities of these T cells are also regulated by the cytokines IL-1, IL-6, IL-8, IL-10, IL22, IL-23, TNF-α and so on^5–8^. Therefore, the expression of cytokines might influence the severity of platelet destruction and vascular endothelial injury. In other words, alterations in the above cytokines may increase the risk of bleeding in ITP patients. Moreover, the risk of bleeding is controlled by a complex regulatory network that is built from a wide diversity of interacting molecular components. Nevertheless, there is still little information on the relevance of these cytokines to the risk of bleeding in ITP patients. Hence, it is necessary to determine the relationships between these cytokines and the risk of bleeding by using a model based on multiple cytokines.

In this study, a cytometric bead array (CBA) human inflammation kit was used to detect the expression of IFN-γ, IL-1, IL-4, IL-6, IL-8, IL-10, IL-17A, IL-22, IL-23, TNF-α and TGF-β. Then, a predictive model was established by combining the expression levels of these cytokines with clinical information from patients. The model was used to determine the relationships between cytokines and bleeding risk. In addition, it is meaningful for clinicians to estimate the bleeding risk of ITP patients.

## Patients and methods

### Patients

Research approval was obtained from Xi Yuan Hospital, China Academy of Chinese Medical Sciences’ Ethics Committee (2015XLA108-2), and all participants provided informed consent in accordance with the relevant regulations and guidelines. Patients in training and validation cohorts were recruited from the Xiyuan Hospital, China Academy of Chinese Medical Sciences and Dongfang Hospital, Beijing University of Chinese Medicine, between January 2016 and January 2019, and they came from all over China. All the patients we enrolled were diagnosed according to Chinese guidelines for treatment of adult primary immune thrombocytopenia^9^: (1) Finding thrombocytopenia during a routine blood count at least twice. Blood flm should be examined to exclude pseudothrombocytopenia and to check for morphological abnormalities of the blood cells. (2) Splenomegaly occurs infrequently in ITP patients. (3) Bone marrow examination is recommended. A bone marrow exam of ITP patients will show a normal or increased megakaryocyte count, with a decreased thromocytogenic megakaryocyte count. (4) Exclusion of secondary thrombocytopenia. The co-morbidity of patients were HT and DM, and there were no patients with other diseases such as strock, heart, and hyperlipidemia. There were no patients used drugs such as antiplatelet, anticoagulant, ant-lipid. The patients enrolled didn’t take any other medication other than glucocorticoid (Prednisone) for treatment of ITP such as thrombopoietin and rituximab. All participating patients provided written informed consent and completed questionnaires including personal information.

### Cytokine analysis

Two milliliter of venous blood was collected from each volunteer, then 200 μl serum were separated by density gradient centrifugation for cytokines analysis. The levels of cytokines (IFN-γ, IL-1, IL-4, IL-6, IL-8, IL-10, IL-17A, IL-22, IL-23,TNF-α and TGF-β) were detected by using a cytometric bead array (CBA) human inflammation kit according to the manufacturer's instructions (BD Pharmingen, San Diego, C A, USA) and analyzed by AimPlex Bead-based multiparametric flow cytometry (EPICS-Elite, Beckman-Coulter, USA). Briefly, AimPlex Bead-based Multiplexed Immunoassays for Flow are similar in principle to a sandwich ELISA, with each bead population conjugated with a specific capture antibody to trap the protein of interest, such as a cytokine, in a sample. The amount of the analyte captured is detected via a biotinylated antibody that recognizes a secondary epitope in the protein, followed by streptavidin-PE treatment. The fluorescence intensity of PE on the beads is quantified on a flow cytometer. The concentration of a protein of interest in a sample can be obtained by comparing the fluorescence signals of the sample to those of a standard curve generated from serial dilutions of a known concentration of the analyte^10,11^.

### Assessment of hemorrhage

The evaluation of bleeding was based on the patient history of bleeding and physical examination during the first week of consultation. The examination covered 9 anatomical sites including the skin, oral cavity, nose, gastrointestinal tract, urinary system, gynecological tract, lungs, intracranial and conjunctiva. Then, patients were divided into two groups according to ITP-BAT score^12^, no bleeding group was 0 score, the others was bleeding group, which score was above 0.

### Statistical analysis

R software was used for statistical analysis (Version 3.5.3; https://www.R-project.org).

The least absolute shrinkage and selection operator (LASSO) method was used to select the optimal predictive features among risk factors for bleeding in patients with ITP because it is fit to constrain high-dimensionality data^13^. Features without nonzero coefficients are excluded in the LASSO regression model^14^. Then, a predictive model was built based on the results of multivariate logistic regression analysis, which also incorporated the results of the LASSO regression model. The odds ratio (OR), 95% confidence interval (CI) and P-value were calculated for all features. The statistical evaluations were all two-sided. Demographic characteristics with a P-value ≤ 0.05 were included in the model, and variables associated with disease or treatment characteristics were also included. All potential predictors were applied to develop a predictive model for the risk of bleeding by using the cohort^15^.

Calibration curves were used to assess the calibration of the bleeding risk nomogram. A significant test statistic would indicate that the model was not perfectly calibrated^16^. Then, the discrimination performance of the bleeding risk nomogram was quantified with the area under the receiver operating characteristic (ROC) curve and Harrell’s C-index. Subsequently, bootstrapping validation (1,000 bootstrap resamples) was conducted to calculate a relatively corrected C-index for the bleeding risk nomogram^17,18^. However, the clinical usefulness of the bleeding risk nomogram was detected by decision curve analysis, which quantified the net benefits at different threshold probabilities in the validation dataset^19^. However, the method of calculating net benefit was to subtract the proportion of all false-positive patients from the proportion of true-positive patients and to weigh the relative risk of intervention against the negative consequences of needless intervention^20^.

## Results

### Patient characteristics

There were 100 patients of training cohort and 88 patients of validation cohort enrolled in this study, included persistent (3–12 months) and chronic (> 12 months) patients with the platelet count range of 12–78 × 10^9^/L. All of the members were divided into bleeding and nonbleeding groups according to ITP-BAT score, no bleeding group was 0 score, the others was bleeding group, which score was above 0. The results for the expression of cytokines are shown for four groups, which were created by grouping by percentile (25%, 50%, and 75%). Additionally, patients were divided in three groups according to the platelet count based on consensus-based recommendation for target platelet counts for surgery or medical therapy in adults^21^. All patients data, including demographic and clinical characteristics, in the two groups are reported in Table 1.

**Table 1:** Characteristics of patients in the training and validation cohorts.

	Training cohortn (%)			Validation cohortn (%)		
	Bleeding	No Bleeding	*P*	Bleeding	No Bleeding	*P*
	n = 65	n = 35		n = 43	n = 45	
**Demographic characteristics**						
Age						
18–60	51 (78.5)	29 (82.9)	0.6	31 (72.1)	39 (86.7)	0.09
≥ 60	14 (21.5)	6 (17.1)		12 (27.9)	6 (13.3)	
Gender						
Male	23 (35.4)	10 (28.6)	0.48	14 (32.6)	15 (33.3)	0.93
Female	42 (64.6)	25 (71.4)		29 (67.4)	30 (66.7)	
Education level						
Primary school	21 (32.3)	10 (28.6)	0.78	13 (30.2)	11 (24.4)	0.75
Middle school	26 (40)	13 (37.1)		14 (32.6)	14 (31.1)	
University	18 (27.7)	12 (34.3)		16 (37.2)	20 (44.4)	
Occupational						
Non-manual labor	40 (61.5)	23 (65.7)	0.68	33 (76.7)	31 (68.9)	0.41
Physical labor	25 (38.5)	12 (34.3)		10 (23.3)	14 (31.1)	
**Clinical characteristics**						
Disease duration (months)						
3–12	11 (16.9)	5 (14.3)	0.73	21 (48.8)	16 (35.6)	0.21
> 12	54 (83.1)	30 (85.7)		22 (51.2)	29 (64.4)	
Comorbidities						
Hypertension	6 (9.2)	3 (8.5)	0.9	3 (7)	1 (2.2)	0.45
Diabetes	1 (1.5)	1 (2.8)		2 (4.7)	1 (2.2)	
No comorbidities	58 (90.3)	31 (89.3)		38 (88.4)	43 (95.6)	
Current use of GC						
Yes	40 (61.6)	21 (60)	0.87	23 (53.5)	20 (44.4)	0.4
No	25 (38.4)	14 (40)		20 (46.5)	25 (55.6)	
PLT (× 10^9^/L)						
< 20	22 (33.9)	3 (8.6)	0.02	21 (48.8)	5 (11.1)	0.01
20–50	29 (44.6)	20 (57.1)		15 (34.9)	30 (66.7)	
51–80	14 (21.5)	12 (34.3)		7 (16.3)	10 (22.2)	
IFN-γ (pg/ml)						
< 4.25	13 (20)	10 (28.6)	0.72	34 (79.1)	28 (62.2)	0.01
4.25–5.02	18 (27.7)	9 (25.7)		6 (14)	2 (4.4)	
5.03–16.4	18 (27.7)	7 (20)		1 (2.3)	8 (17.8)	
> 16.4	16 (24.6)	9 (25.7)		2 (4.7)	7 (15.6)	
IL-1β (pg/ml)						
< 1.57	15 (25.1)	10 (28.6)	0.48	22 (51.2)	24 (53.3)	0.92
1.57–2.84	18 (27.7)	6 (17.1)		11 (25.6)	13 (28.9)	
2.85–125.31	18 (27.7)	8 (22.9)		3 (7)	2 (4.4)	
> 125.31	14 (21.5)	11 (31.4)		7 (16.3)	6 (13.3)	
IL-4 (pg/ml)						
< 3.17	19 (29.2)	5 (14.3)	0.36	12 (27.9)	7 (15.6)	0.37
3.17–3.71	15 (25.1)	11 (31.4)		7 (16.3)	6 (13.3)	
3.72–12.59	14 (21.5)	10 (28.6)		21 (48.8)	30 (66.7)	
> 12.59	17 (26.2)	9 (25.7)		3 (7)	2 (4.4)	
IL-6 (pg/ml)						
< 6.02	15 (25.1)	10 (28.6)	0.26	28 (65.1)	25 (55.6)	0.44
6.02–8.46	19 (29.2)	6 (17.1)		2 (4.7)	5 (11.1)	
8.47–9,855.8	18 (27.7)	7 (20)		7 (16.3)	11 (24.4)	
> 9,855.8	13 (20)	12 (34.3)		6 (14)	4 (8.9)	
IL-8 (pg/ml)						
< 19.17	20 (30.8)	5 (14.3)	0.31	15 (34.9)	13 (28.9)	0.57
19.17–41.49	14 (21.5)	10 (28.6)		18 (41.9)	16 (35.6)	
41.5–44,418.72	16 (24.6)	9 (25.7)		8 (18.6)	11 (24.4)	
> 44,418.72	15 (25.1)	11 (31.4)		2 (4.7)	5 (11.1)	
IL-10 (pg/ml)						
< 3.99	14 (21.5)	11 (31.4)	0.73	4 (9.3)	4 (8.9)	0.46
3.99–4.75	16 (24.6)	8 (22.9)		17 (39.5)	11 (24.4)	
4.76–12.98	19 (29.2)	8 (22.9)		21 (48.8)	28 (62.2)	
> 12.98	16 (24.7)	8 (22.9)		1 (2.3)	2 (4.4)	
IL-17A (pg/ml)						
< 143.65	20 (30.8)	5 (14.3)	0.06	6 (14)	7 (15.6)	0.42
143.65–179.38	17 (26.2)	8 (22.9)		5 (11.6)	11 (24.4)	
179.39–480.2	17 (26.2)	8 (22.9)		29 (67.4)	25 (55.6)	
> 480.2	11 (16.8)	14 (40)		3 (7)	2 (4.4)	
IL-22 (pg/ml)						
< 5.26	20 (30.8)	5 (14.3)	0.12	8 (18.6)	11 (24.4)	0.84
5.26–6.8	18 (27.7)	7 (20)		17 (39.5)	14 (31.1)	
6.81–2,743.68	14 (21.5)	11 (31.4)		16 (37.2)	18 (40)	
> 2,743.68	13 (20)	12 (34.3)		2 (4.7)	2 (4.4)	
IL-23 (pg/ml)						
< 2.43	16 (24.6)	9 (25.7)	0.3	11 (25.6)	15 (33.3)	0.83
2.43–3.24	20 (30.8)	5 (14.3)		12 (27.9)	11 (24.4)	
3.25–340.81	14 (21.5)	11 (31.4)		8 (18.6)	9 (20)	
> 340.81	15 (25.1)	10 (28.6)		12 (27.9)	10 (22.2)	
TNF-α (pg/ml)						
< 2.24	19 (29.2)	5 (14.3)	0.29	13 (30.2)	12(26.7)	0.49
2.24–2.99	15 (25.1)	11 (31.4)		16 (37.2)	14(31.1)	
3–115.84	17 (26.2)	8 (22.9)		11 (25.6)	11(24.4)	
> 115.84	14 (21.5)	11 (31.4)		3 (7)	8(17.8)	
TGF-β (pg/ml)						
< 6,959.06	20 (30.8)	6 (17.1)	0.05	14 (32.6)	11(24.4)	0.01
6,959.06–12,480.82	18 (27.7)	7 (20)		19 (44.2)	5(11.1)	
12,480.83–67,139.67	17 (26.2)	8 (22.9)		8 (18.6)	15(33.3)	
> 67,139.67	10 (15.3)	14 (40)		2 (4.7)	14(31.1)	

*GC* glucocorticoid (Prednisone).

### Feature selection

**Figure 1:**
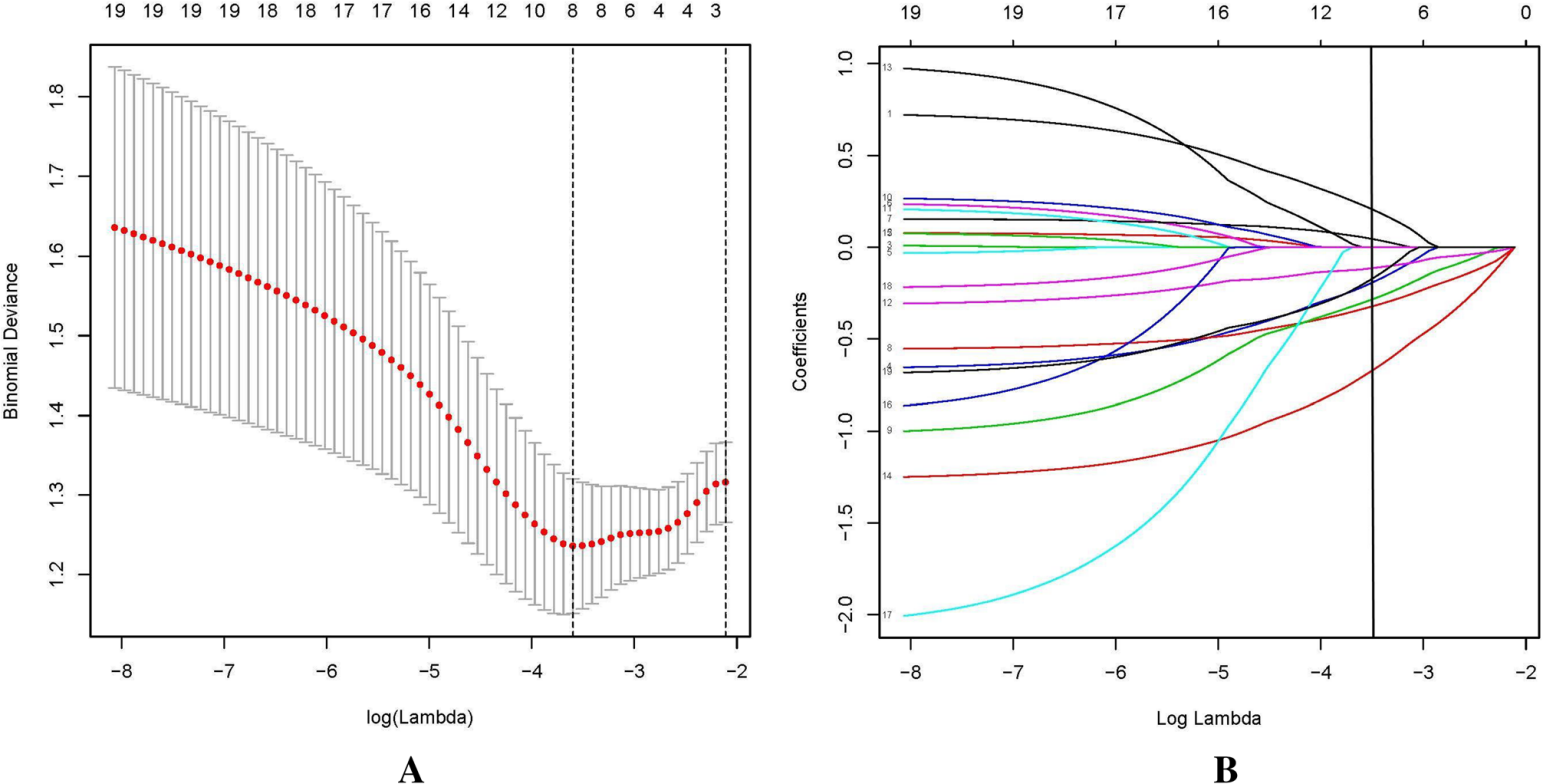
Results for demographic and clinical feature selection by the LASSO binary logistic regression model. *Notes* The optimabest parameter (lambda) of the lasso model, which is selected by the minimum criterion for five cross verifications, shown in (**A**). The binomial deviance curve was plotted depended on log(lambda). According to the minimum criteria and the 1-SE criteria, dotted vertical lines were drawn at the optimal values. (**B**) Showed LASSO coefficient profiles of the 19 features. A coefficient profile plot was produced based on the log(lambda) sequence. Vertical line was drawn at the value selected using fivefold cross-validation, where optimal lambda resulted in eight features with nonzero coefficients. *LASSO* least absolute shrinkage and selection operator, *SE* standard error.

The 19 features (showed in Table 1) of 100 patients were reduced to eight potential predictors based on the LASSO regression model. Dotted vertical lines were drawn at the optimal values by using the minimum criteria and the 1 standard error of the minimum criteria (the 1-SE criteria). A lambda value of 0.097, with log (lambda),-3.37 was chosen (1-SE criteria) according to fivefold cross-validation (Fig. 1A,B). These features included the expression of IFN-γ, the expression of IL-4, the expression of IL-10, the expression of IL-17A, the expression of IL-22, the expression of TGF-β, the count of PLT and the length of time of ITP (Table 2).Then, logistic regression also performed for further verification. The results were showed in Table 2. Except PLT and IL-17A, the p value of the other 6 features are blew 0.05. However, PLT and IL-17A are meaningful to ITP, we still use the above 8 features for further forecasting.

**Table 2:** Predictive factors for bleeding risk in ITP.

Intercept and variable	Prediction model		
	β	Odds ratio (95% CI)	*P*-value
IFN-γ	4.71284305	2.88 (0.569, 16.854)	0.00214
IL-4	− 2.35877965	0.41 (0.06, 2.39)	0.03499
IL-10	− 2.16067288	0.115 (0.126,4.54)	0.03162
IL-17A	0.65924361	1.933 (0.094,4.167 )	0.45221
IL-22	− 1.77017665	0.17 (0.033, 1.534)	0.04587
TGF-β	− 2.03386875	0.131 (0.142, 4.451)	0.06987
PLT	− 1.18593598	0.305 (0.045, 1.227)	0.25476
Time	− 0.75794663	0.469 (0.133, 1.513)	0.02765

β is the regression coefficient of eight feathers enrolled in logistic regression model.

If β is coefficient is positive and odds ratio is above one, the feature is positively correlated with the probability of occurrence of bleeding. If β is coefficient is negative and odds ratio is below one, the feature is positively correlated with the probability of occurrence of no bleeding.

### Prediction model

**Figure 2:**
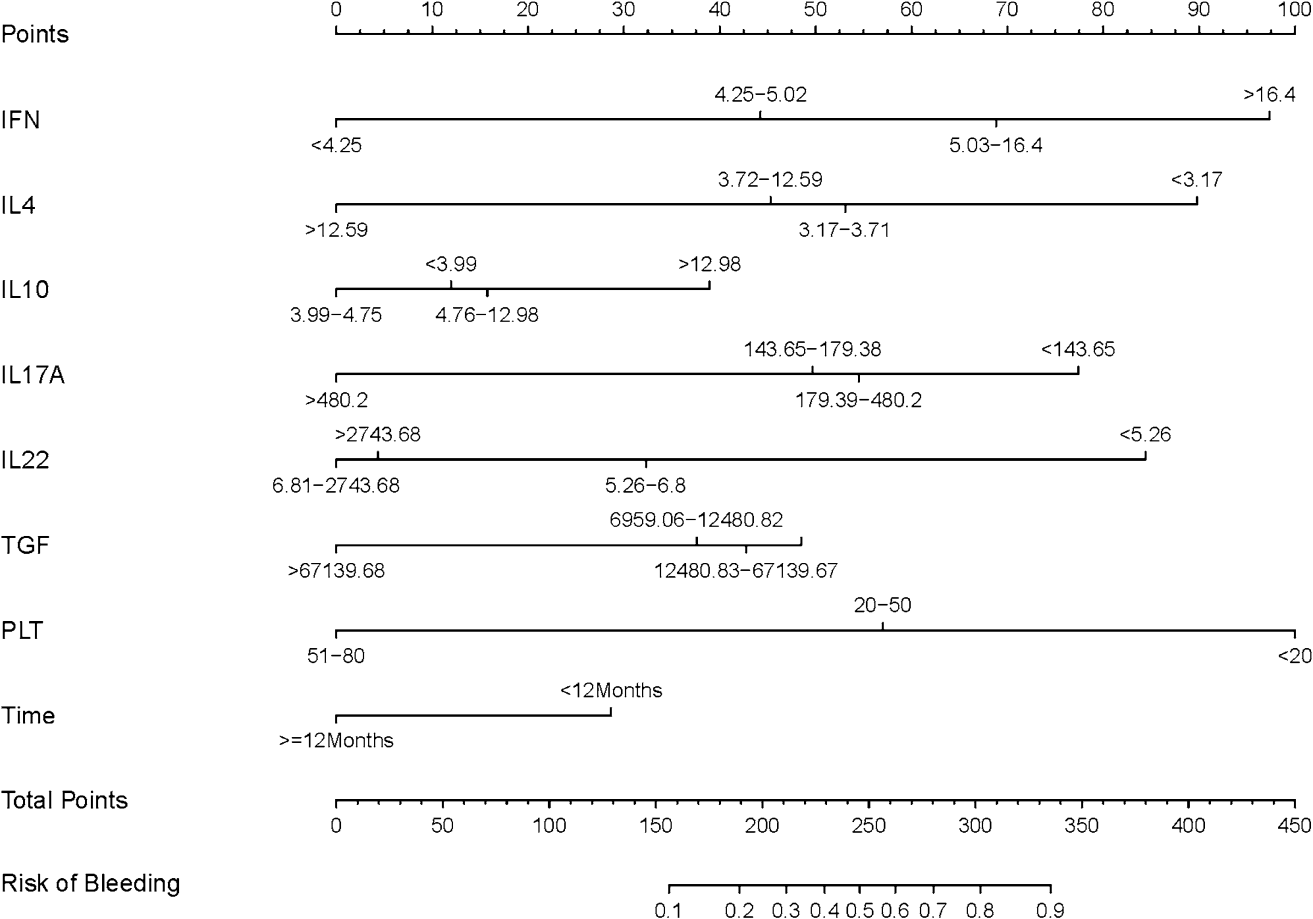
The nomogram of bleeding risk predict model. *Note* To use the nomogram, an individual patient’s value is located on each variable axis, and a line is drawn upward to estimate the number of points received for each variable value. The sum of these numbers is located on the Total Points axis, and a line is drawn downward to the survival axes to determine the likelihood of bleeding.

According to the results of the LASSO regression analysis, 8 potential predictors were selected and analyzed by logistic regression for further forecasting. The results are shown in Table 2. Then, the forecast model that included the above independent predictors was developed and is presented as the nomogram (Fig. 2).

### Accuracy of the bleeding risk nomogram

**Figure 3:**
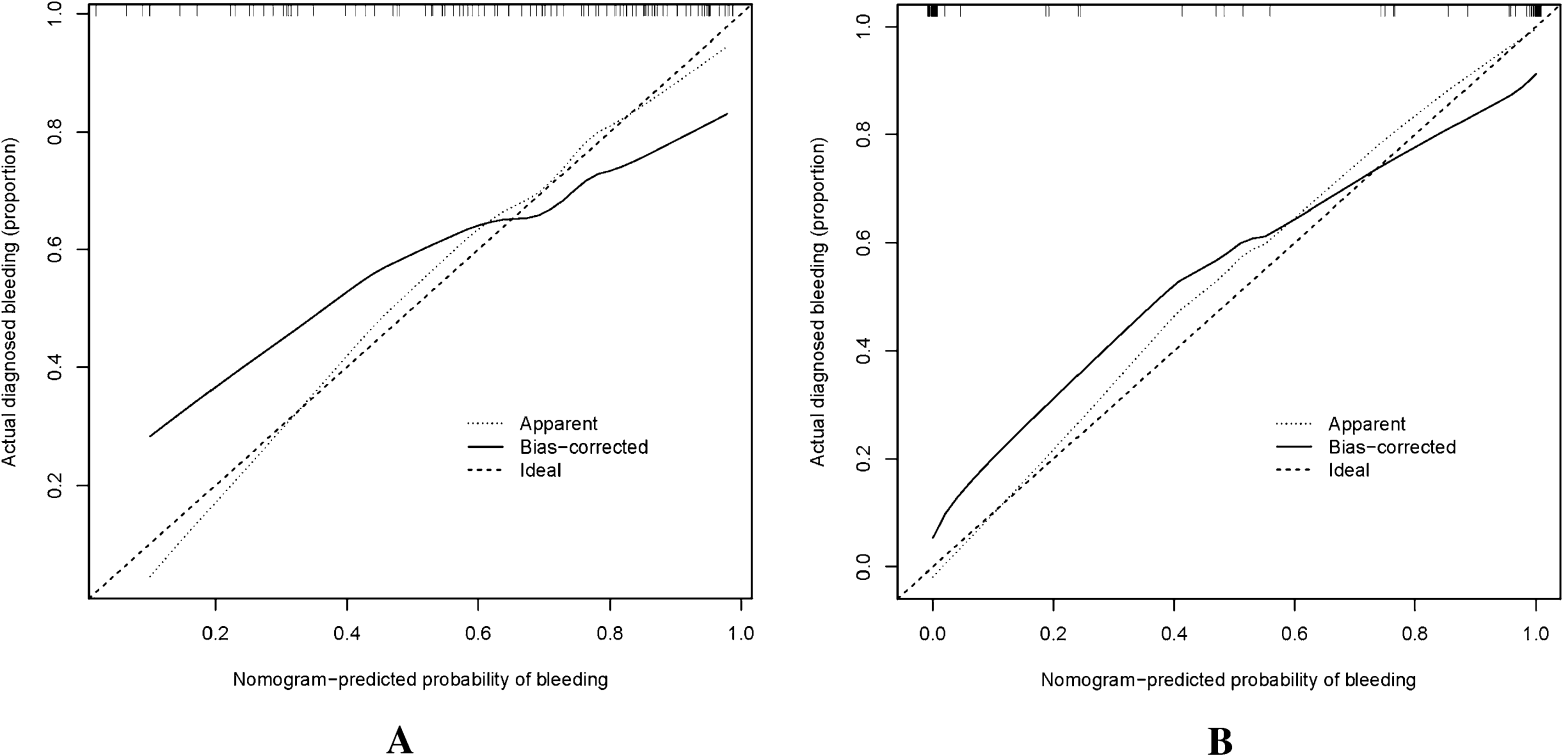
Calibration curves for bleeding nomogram predictions in the training cohort (**A**) and Validation cohort (**B**). *Notes* The x-axis represents the forecasted bleeding risk, while the the actual diagnosed bleeding shown at y-axis. The diagonal dotted line showed an ideal model for the perfect prediction ability, and the solid line (bias-corrected line) represents the reality performance of the nomogram. The closer fit to the diagonal dotted line, the better prediction ability of the nomogram.

**Figure 4:**
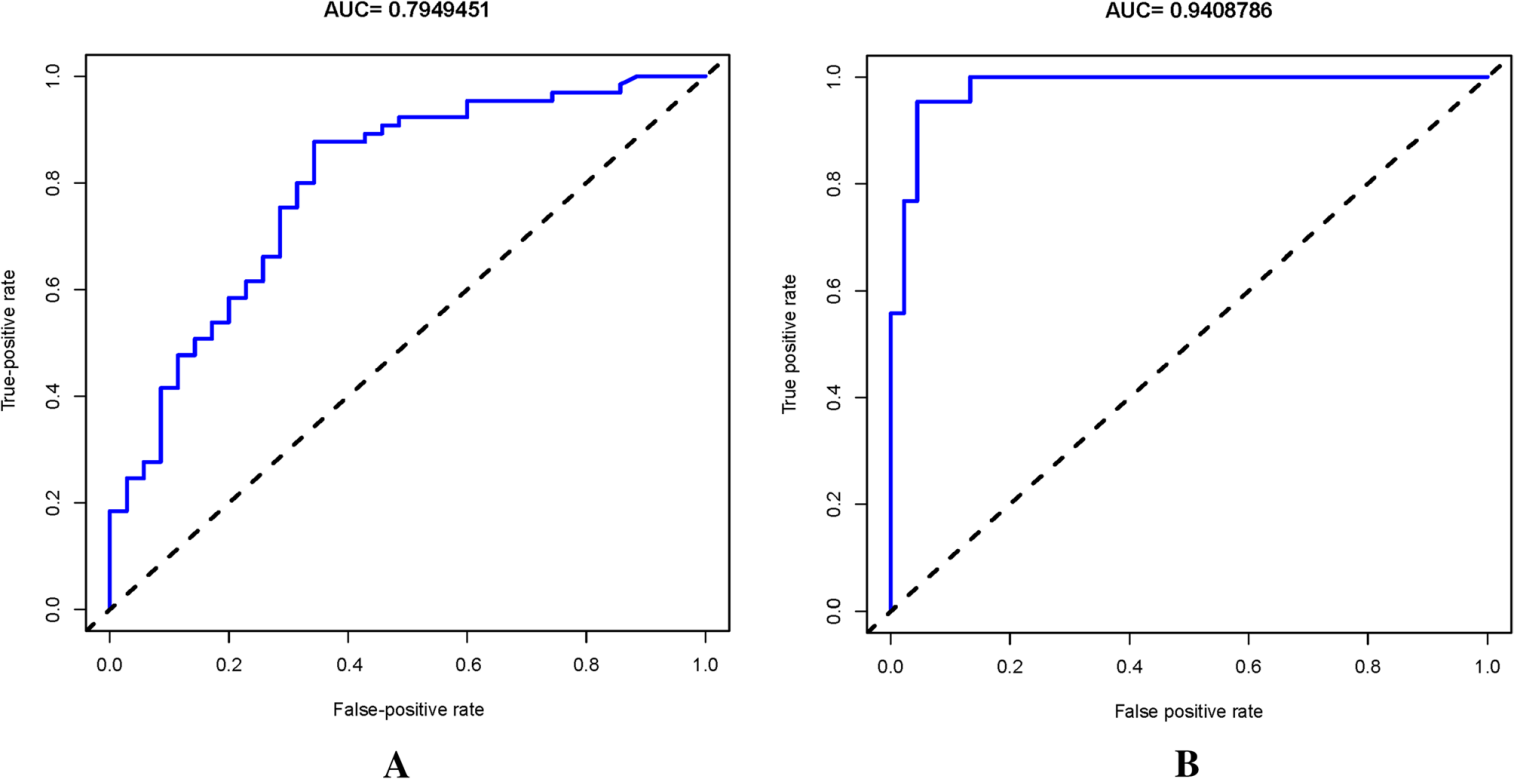
ROC curves of bleeding nomogram in the training cohort (**A**) and validation cohort (**B**). *Notes* The y-axis represents true positive rate and x-axis shows false positive rate. The area between dotted line and the curve is AUC, and the larger of AUC (closed to one), is the higher of model’s accuracy. Then, the accuracy of bleeding risk model shows great based on AUC.

The nonadherence nomogram was subjected to internal verification and external verification. The calibration curve of the bleeding risk nomogram for the prediction of bleeding risk in ITP patients demonstrated good agreement in training cohort and validation cohort (Fig. 3). The C-index for the predictive nomogram was 0.82 (95% CI 0.73376–0.90624) for the training cohort and was confirmed to be 0.6689 through internal bootstrapping validation and 0.89(95% CI 0.868, 0.902)in validation cohort, which suggested that the model had good discrimination. In addition, the AUC for the predictive nomogram was 0.79 in training cohort and 0.94 in validation cohort (Fig. 4), which also showed that the model had a good prediction ability. In the bleeding risk nomogram, apparent performance addressed the good prediction capability.

### Clinical application

**Figure 5:**
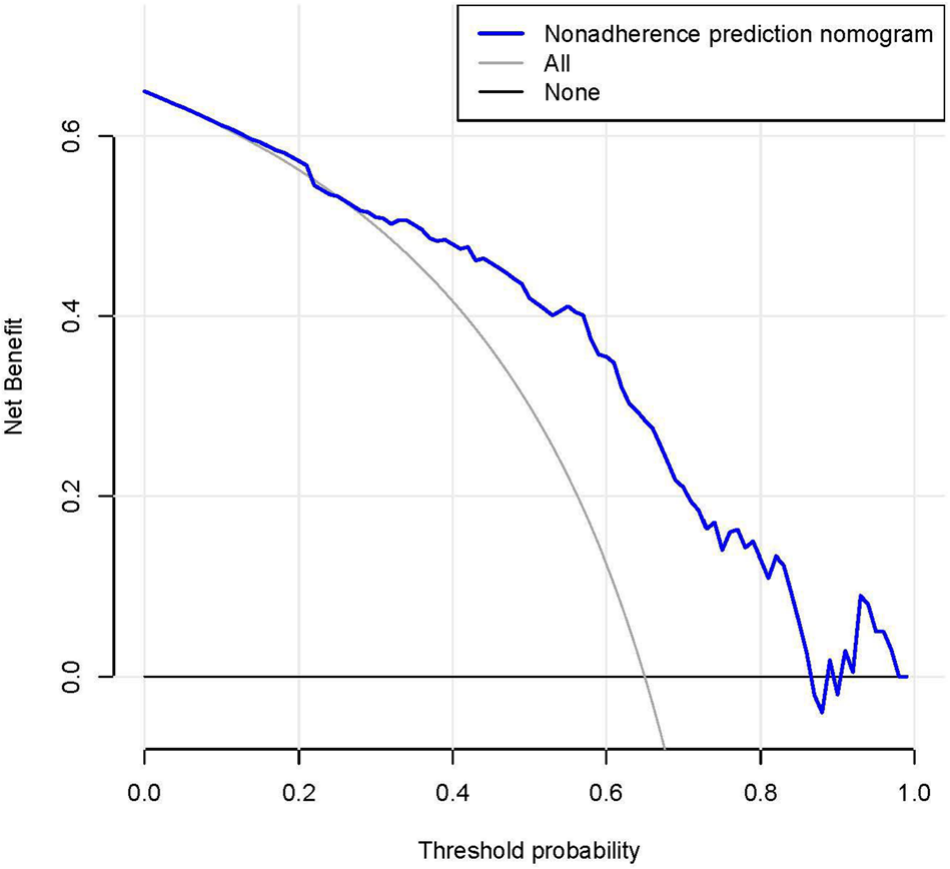
Decision curve analysis for the bleeding risk nomogram. The y-axis measures the net benefit. The blue line represents the bleeding risk nomogram. The thin solid line represents the assumption that all patients were bleeding during the course of ITP progression. The thick solid line (parallel to the x-axis) represents the assumption that no patients were bleeding. The net benefit was calculated by subtracting the proportion of all patients who are false positive from the proportion who are true positive, weighting by the relative harm of forgoing treatment compared with the negative consequences of an unnecessary treatment. In this study, 14% (the intersection of blue line and thin solid line) was false positive rate and 88% (the intersection of blue line and thick solid line) was false negative rate.

Decision curve analysis of the bleeding risk nomogram is presented in Fig. 5. The decision curve showed that if the threshold probability of a patient was > 14 and < 88%, using this bleeding risk nomogram to predict bleeding risk added more benefit than the scheme did. Within this range, using the bleeding nomogram developed in the current study to predict bleeding risk added more benefit than the intervention-all-patients scheme or the intervention-none scheme.

## Discussion

Currently, risk nomograms are widely used as prognostic models for clinical decision-making. For ITP patients, bleeding risk still remains an enormous burden that affects quality of life. Although compared with patients with other hemorrhagic diseases, the majority of ITP patients have relatively mild bleeding symptoms, an objective and accurate description is still needed to study the heterogeneity of bleeding tendency in this disease. However, a useful model is still lacking for assessing bleeding risk. Therefore, it is necessary to construct a risk nomogram that can be used by clinicians to clarify and control the risk of bleeding.

However, ITP is considered to be a consequence of complex immunoregulation disorder events in T cells, B cells and related cytokines. Previous studies showed that the development of ITP was connected with immunoregulation disorder caused by Th1/Th2 and Th17/Treg cell biases and abnormal secretion by Bregs^22–24^. Th cells play a central r ole in the maintenance of the immune balance. The Th1/Th2 and Th17/Treg cytokine ax es are closely associated with autoimmunity. Upon antigen stimulation, CD4 + T cells can differentiate into at least four functional subtypes (Th1, Th2, Th17 and Treg), which have unique patterns of effector cytokine secretion^2^. Th1, Th2, Th17 and Treg cells are differentiated from Th0 cells. Th1 cells secrete the specific cytokine IFN-γ, Th2 cells secrete the specific cytokine interleukin-4 (IL-4), T helper 17 (Th17) cells secrete the specific cytokine IL-17A, and Treg cells secrete the specific cytokine TGF-β. Breg cells are another kind of immunoregulatory cell that secretes the specific cytokines IL-10 and IL-22^25–27^.

Generally, cytokines can be divided into two types depending on their function. One type is proinflammatory, and the other is anti-inflammatory. The expression of proinflammatory and anti-inflammatory factors tends to be balanced under physiological conditions; otherwise, immune disorders occur.

Additionally, cytokines are derived from immune cells, and the secretion and function of immune cells are regulated by related cytokines. In other words, the occurrence and development of ITP is due to the overexpression of proinflammatory factors and low expression of anti-inflammatory factors, according to research^28^.

Since cytokines are so important in the onset of ITP, cytokines should be incorporated into bleeding models. However, previous hemorrhagic risk assessment models did not take cytokines into account. In this study, a relatively accurate predictive tool was used to evaluate bleeding risk in patients with ITP. The internal validation and external validation of the tool showed good discrimination and calibration capabilities; in particular, our high c-index measured by interval validation and AUC indicate that our model can be used widely and accurately to assess bleeding risk.

Regarding the risk of bleeding in ITP patients, a previous studies mainly focused on the effect of platelets, including the platelet count^29^, platelet function^30^, platelet activation^31^, and the immature platelet fraction^32^. However, these models, such as the ITP-BAT^33^, WHO bleeding scoring system^34^, Buchanan hemorrhage score^35^, and Khellaf hemorrhage score^36^, are based on only clinical hemorrhagic symptoms. It is difficult to assess the risk of bleeding in a patient if he or she bled previously, but the symptoms have disappeared or no positive signs of bleeding are found on physical examination. At this time, the risk of bleeding is underestimated, which affects treatment choice. Additionally, low platelet counts do not always indicate a high bleeding risk; therefore, if treatment is based only on platelet counts, overtreatment may occur, resulting in wasted medical resources and forcing patients to take unnecessary medical risks. In addition, platelet function detection methods include optical turbidimetry, the impedance method, thromboelastography, Plateletwork tests and flow cytometry^37,38^. However, these methods have some defects, such as poor replicability, a requirement for a high platelet quantity and expensive instruments. In fact, predicting the bleeding risk in individual patients is difficult, and the inclusion of various factors and multifaceted interventions may be the most effective solution. Therefore, it is very necessary to find an index that can comprehensively evaluate the hemostatic state of ITP patients while also being convenient and inexpensive. Similar to previous studies, our study showed that a low platelet count and long course of disease increased the risk of bleeding. In contrast to previous studies, this study took into consideration the expression of cytokines.

In this study, 65% of ITP patients exhibited bleeding, including in the purpura, muscle, nose, retinal and gingival. Then, we obtained eight key factors that forecasted the risk of bleeding in ITP patients by risk factor analysis. This nomogram suggested that patients with high expression of IFN-γ, IL-17A, and IL-22; low expression of IL-4, IL-10, and TGF-β; a low platelet count, and a long course of disease had an elevated risk of bleeding. This result basically agreed with clinical feature findings. Therefore, it is helpful to take into account cytokines when predicting bleeding risk. This model will assist physicians in accessing the bleeding risk of ITP patients and taking interventions in time, preventing unnecessary measures in low-risk situations and avoiding delays or discontinuity in treatment when the most appropriate time appears. We also believe that this model will be beneficial in the evaluation of drug efficacy since improvements in patients with bleeding symptoms are as important as improvements in platelet levels.

## Limitations

There are also several limitations of our current study. First, our study did not enroll newly diagnosed patients (less than 3 months) and the platelet level as 80–< 100 were not included, so the bleeding risk for these patients cannot be evaluated. Second, children and teenagers were not included, so the difference between pediatric and adult patients was not be taken into consideration. Third, the cohort was not representative of all Chinese patients with ITP since patient race and region of origin were not considered. These factors need to be externally evaluated in a broader population of ITP patients.
